# Hydrogen Gas Fumigation Combined with Nano-Film Packaging Extend the Storage of Button Mushrooms (*Agaricus bisporus*)

**DOI:** 10.3390/foods14060952

**Published:** 2025-03-11

**Authors:** Jiawei Shen, Yajie Zhang, Biao Wang, Wenwei Zhang, Liang Yao, Jianmin Yun

**Affiliations:** 1 College of Food Science and Engineering, Gansu Agricultural University, Lanzhou 730070, China; m19935452472@163.com (J.S.); zhangyajie1302@163.com (Y.Z.); zhangww@gsau.edu.cn (W.Z.); 2 Qingyang Agricultural Technology Promotion Centre, Qingyang Agricultural and Rural Bureau, Qingyang 745000, China; wangbiao02299@163.com; 3 Gannong Moli (Qingyang) Agricultural Development Co., Ltd., Qingyang 745000, China; 19935452769@163.com

**Keywords:** hydrogen gas fumigation, polyethylene nano-packaging, *Agaricus bisporus*, freshness-keeping effect, antioxidant capacity

## Abstract

To extend the shelf life of button mushrooms, the optimal fumigation doses for hydrogen gas treatment were screened through sensory evaluation, combined with browning index and weight loss rate in this study. Then, using H_2_ fumigation combined with polyethylene film packaging as a control, changes in the sensory quality, reactive oxygen species, browning-related enzyme activity and the nutritional quality of mushrooms treated by H_2_ fumigation combined with nano-film packaging (H_2_ + NA) during low-temperature storage were dynamically tracked. The preservation effect of H_2_ + NA on mushrooms after harvest was investigated, and its mechanism was also analyzed. The storage validation test showed that the optimum H_2_ fumigation time was 2 h, and the H_2_ + NA-treated mushrooms had a fuller appearance, maintained whiteness well, showed a slow increase in reactive oxygen species, antioxidant enzyme activities remained at high levels, a high retention rate of protein content was observed, and there was a good antibacterial effect. This study indicates that H_2_ fumigation combined with nano-film packaging can improve the storage quality of button mushrooms and may prolong low-temperature shelf life by 4–5 d compared to conventional commercial polyethylene film packaging.

## 1. Introduction

Button mushrooms have become the most widely consumed edible mushroom in the world due to their white body, crispy texture, and high nutritional, pharmaceutical, and antioxidant capacity [1]. Usually, button mushrooms are sold as fresh products [2]; however, they possess a short shelf life owing to their high moisture content and lack of a protective layer, leading to browning and softening, which largely reduce their appearance quality, edible value, and commercial value [3].

Studies have shown that some modified atmosphere packaging (MAP) approaches have been proven to effectively prevent deterioration in mushroom quality [4], such as 30% O_2_ and 70% N_2_ [5] and 30% CO_2_ and 70% N_2_ [6]. However, excessive use of CO_2_ may lead to a decline in the quality of some foods and pose a threat to the environment. Thereby, the development of novel composite preservation technology is of great significance for maintaining the quality and prolonging the shelf life of *A. bisporus*.

Hydrogen (H_2_) is a colorless odorless gas, and as a typical reducing agent, it has been applied in medicine and plant adversity defense [7]. Studies have shown that H_2_ has antioxidant, anti-inflammatory, and anti-apoptotic activities [8], and the antioxidant effect of H_2_ can significantly attenuate the oxidative stress of agricultural products [9,10]. Additionally, Liu et al. [11] found that H_2_ acted as a signaling molecule participating in the defensive reaction of plants to diverse abiotic stresses. Some studies have found that H_2_ fumigation can delay the postharvest quality deterioration in lilies and reduce the decline in nutritional quality [12], can reduce the decay rate in Chinese chives [13], and effectively extend the shelf life of food [14,15]. Nevertheless, scant reports exist regarding the impacts of H_2_ fumigation on the postharvest storage quality and antioxidant capacity of mushrooms.

Nanomaterials, as cutting-edge and interdisciplinary emerging materials, are becoming a popular field of research, and are characterized by antioxidant activity, microbial growth inhibition, being a gas and moisture barrier [16,17], and having unique properties that traditional packaging does not have, and thus are widely used in the field of fruit and vegetable preservation [18]. The composite preservation technology of nano-film packaging has the characteristics of low cost, strong operability, high safety, and good preservation effect. It has become an important trend in the development of edible mushroom preservation technology and has extensive application prospects in postharvest preservation of fruit and vegetables, attracting widespread attention from researchers [16,19].

Consequently, this study aimed to investigate the effects of H_2_ fumigation combined with nano-film packaging on the quality and antioxidant capacity of fresh mushrooms during 15 d of cold storage and its mechanism, with a view to providing methodological references for the reduction of loss, preservation of quality, and prolongation of shelf life of *A. bisporus* in postharvest storage and circulation.

## 2. Materials and Methods

### 2.1. Materials

Button mushrooms (*A. bisporus,* A15) were collected from Gannong Moli (Qingyang) Agricultural Development Co., Ltd. (Qingyang, China). After harvest, the mushrooms were transported to the laboratory by the cold chain.

The nano-packaging material (NA) was polyethylene (PE) film containing nanosilver particles (FMSXPBC, Shanghai Fuming New Material Technology Co., Ltd., Shanghai, China). Physical properties of NA were as follows: thickness was 0.03–0.04 mm; the transmission rate for O_2_ was 9000–10,000 cm^3^ m^−2^ 24 h^−1^ at 0.1 MPa; water vapor transmission rate was 23 g m^−2^ h^−1^; good transparency and anti-fogging; tensile strength: longitudinal 38.93 MPa, transverse 33.83 MPa; elongation at break: longitudinal 618%, transverse 799%.

### 2.2. Screening of the Optimum Time for Hydrogen Fumigation

The H_2_ generated by a hydrogen generator (RX-H500, Shanghai Analytical Instrument Co., Ltd., Shanghai, China) was used for fumigation over 0 h, 1 h, 2 h, and 3 h; the mushrooms were packed into plastic boxes with nano packaging; and then the samples were placed in cold storage for 15 d. During the 15 d storage period, the sensory scores, browning index (BI), and weight loss of the treated samples were determined at 3 d intervals. Each determination was replicated three times.

### 2.3. Hydrogen Fumigation Treatment and Packaging of Mushroom Samples

**Figure 1:**
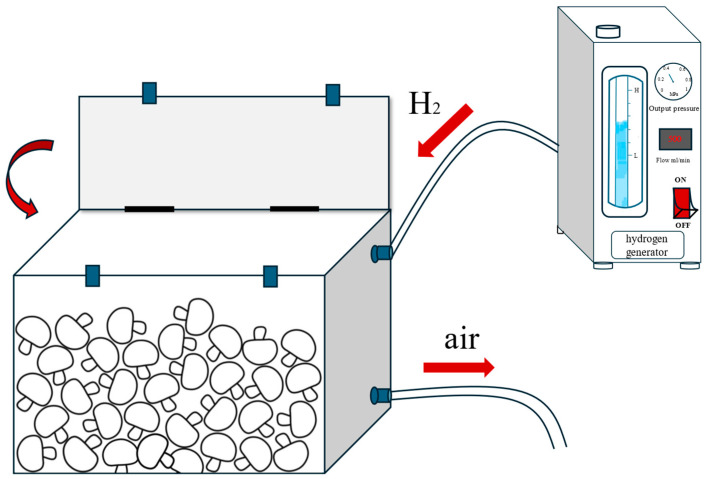
Hydrogen fumigation treatment device.

Based on the pre-screened optimal treatment conditions, the button mushrooms were subjected to hydrogen fumigation treatment. Approximately 10 kg of fresh mushrooms were placed into a 30 L hydrogen fumigation treatment device (as shown in Figure 1), and the hydrogen generator (500 mL/min) was turned on for 45 min to achieve a hydrogen content of about 75% in the container. The gas production was stopped, and the timing was maintained during a particular duration. The mushrooms were then randomly packed in plastic trays, 6 per box (about 200–250 g), with ordinary PE cling film and NA film for packing and sealing (recorded as H_2_ + PE and H_2_ + NA, respectively), and with only PE film and NA film packing as the control group (recorded as CK + PE and CK + NA, respectively), and 3 replicates were included for each treatment (72 boxes in total). The samples were stored in cold storage (temperature: 4 ± 1 °C, relative humidity 90%, volume 20.5 m^3^) for 15 d, and samples were taken every 3 d for measurement.

### 2.4. Sensory Quality Evaluation

The method of Lin et al. [20] was used and modified for sensory evaluation. Five key attributes, namely, color (S1), cap morphology (S2), odor (S3), texture (S4), and consumer acceptance (S5), were selected and evaluated by 10 professionally trained persons. The sensory score = S1 + S2 + S3 + S4 + S5, and the sensory score of each sample was averaged. Fresh *A. bisporus* was used as the control group (sensory score of 10). The organoleptic evaluation criteria are shown in Table 1.

### 2.5. Determination of Weight Loss and Firmness

Weight loss was determined by the gravimetric method as illustrated by Li et al. [21] and stated as a percentage loss of the starting mass of fresh mushrooms. Firmness was determined using a texture analyzer (SMS Co., Godalming, UK) and the method by Kotwaliwale et al. [22].

### 2.6. Determination of the Color and Browning Index (BI)

Adopting the method proposed by Zheng et al. [23] while making certain modifications, a high-precision intelligent spectrophotometer (NS800, Shenzhen Three NH Technology Co., Ltd., Shenzhen, China) was used to determine the L* value (which is used to indicate the whiteness) and BI. The optical density of each mushroom was determined three times following the different treatments, and the brightness (L*), redness (a*), and yellowness (b*) were recorded. The BI was calculated as follows:

(1) $$BI=\frac{100(x-0.31)}{0.17}$$

(2) $$x=\frac{\left(a*+1.75\;L*\right)}{\left(5.645\;L*\right)+(a*-3.012\;b*)}$$

### 2.7. Determination of the Malondialdehyde (MDA) Content

The measurement of MDA content was carried out by utilizing an assay kit (Solarbio, Beijing, China) in line with the manufacturer’s instructions.

### 2.8. Determination of the Content of Reactive Oxygen Species (ROS)

The rate of O^2•−^ production and H_2_O_2_ content were measured using an assay kit (Solarbio, Beijing, China).

### 2.9. Determination of Antioxidant Enzyme Activity

The activities of superoxide dismutase (SOD), and catalase (CAT) were measured using an assay kit (Boxbio, Beijing, China).

### 2.10. Determination of Total Phenols and Flavonoids

A 1 g quantity of mushroom sample was weighed, 10 mL of 80% ethanol was added, and the sample was extracted by ultrasonic extraction at 60 °C for 60 min, and centrifuged at 8000× *g* and 25 °C for 10 min. The supernatant in an amount of 1 mL was taken out and combined with 1 mL of Folin–Ciocalteu reagent and 10 mL of 7% sodium carbonate. Then, distilled water was used to bring the volume of the supernatant up to 25 mL. The absorbance was measured at 760 nm using the Folin–Ciocalteu method [24], and a standard curve was prepared with gallic acid to calculate the total phenol content of sample.

The flavonoid content was assessed using the NaNO_2_-Al (NO_3_)_3_ colorimetric method [25] with some modifications. A 1 g mushroom sample was weighed, 10 mL of 80% ethanol was added, and the sample was extracted by ultrasonic extraction at 60 °C for 60 min, and then centrifuged at 8000× *g* at 25 °C for 10 min. A 2 mL quantity of supernatant was taken and placed in a 10 mL volumetric flask and diluted to 6 mL with 95% ethanol. Subsequently, 0.6 mL of 5% NaNO_2_ test solution was added, and the mixture was shaken well and allowed to stand for 6 min, then mixed with 0.4 mL of 5% Al(NO_3_)_3_. After 6 min, 3.0 mL of 4% NaOH was added; then, it was diluted to the mark with 95% ethanol, shaken well, and incubated for 12 min. The absorbance value was measured at 510 nm, and the flavonoid content (mg/g) of the samples was calculated using the standard curve of rutin.

### 2.11. Determination of Total Aerobic Plate Count

A 10 g quantity of fruiting body samples of *A*. *bisporus* were placed into 90 mL of sterile normal saline. Then, the mixture was oscillated and cultured at 120 r/min and 37 °C for 30 min to prepare a bacterial suspension with a concentration of 10^−1^ CFU/g. We referred to GB4789.2-2022 “National Food Safety Standard Food Microbiological Examination: Determination of Total Aerobic Plate Count” to determine the total number of epiphytic bacteria colonies of *A*. *bisporus*.

### 2.12. Determination of Proteins

The protein content was determined by the Kjeldahl method with reference to GB5009.5-2016 “National Standard for Food Safety: Determination of Protein in Foods”, using a fully automatic Kjeldahl nitrogen analyzer (K9840 Automatic Kjeldahl Nitrogen Analyzer, Shandong Jinan Haineng Instrument Co., Jinan, China).

### 2.13. Statistical Analysis

Measurement of each indicator was repeated three times. Subsequently, the results were presented in the form of mean ± standard deviation, and the obtained data were further analyzed for significance and correlation using IBM SPSS 26.0 software with one-way analysis of variance (ANOVA), in which Duncan’s multiple range test (*p* < 0.05) was used to test the significance of the difference and plotted using Origin 2018.

## 3. Results

### 3.1. Results of Screening the Optimal Time of Hydrogen Fumigation

**Figure 2:**
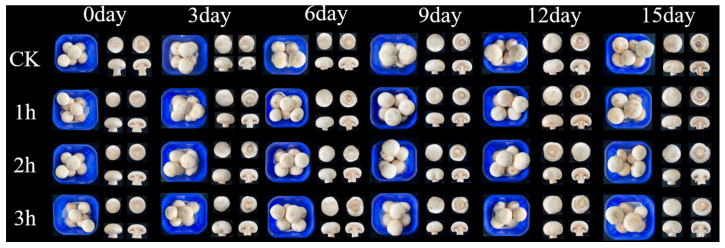
Effects of H_2_ fumigation at different times combined with NA packaging on appearance.

As shown in Table 2 and Figure 2, after H_2_ fumigation for various times, the quality of mushrooms progressively declined as the storage time elapsed, the sensory score gradually decreased, and the BI and weight loss rate increased. Compared with the CK, H_2_ fumigation treatment effectively deferred the browning of mushrooms and diminished the rate of weight loss. During storage, the group of 2 h H_2_ fumigation was at a high level in terms of sensory quality, and at 15 d, the sensory score, weight loss rate, and BI were 44.41, 28.78, and 2.62%, respectively, which were higher than other groups. Therefore, H_2_ fumigation for 2 h was selected for subsequent storage experiments.

### 3.2. Effects of H_2_ + NA on Storage Quality and Overall Acceptability of A. bisporus

**Figure 3:**
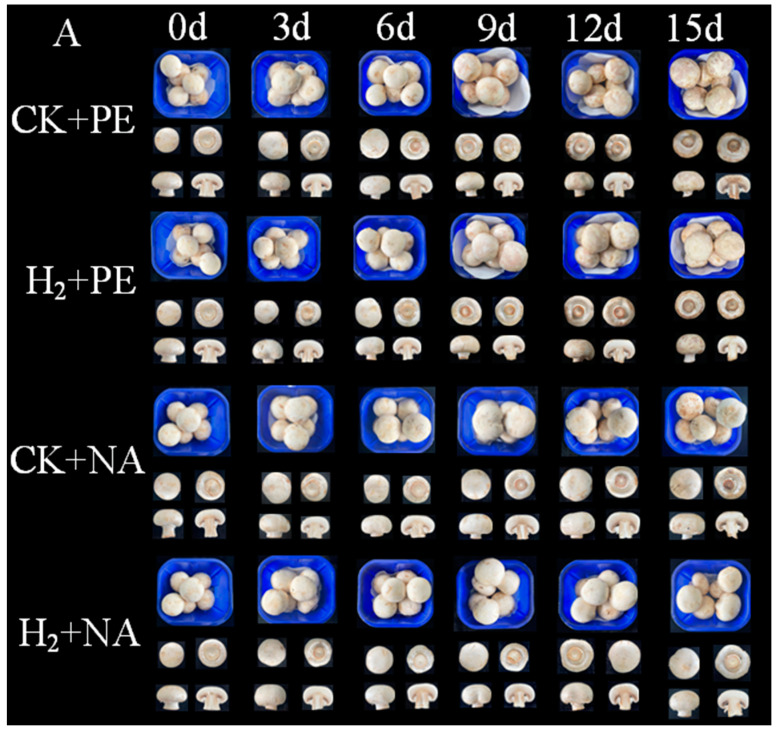

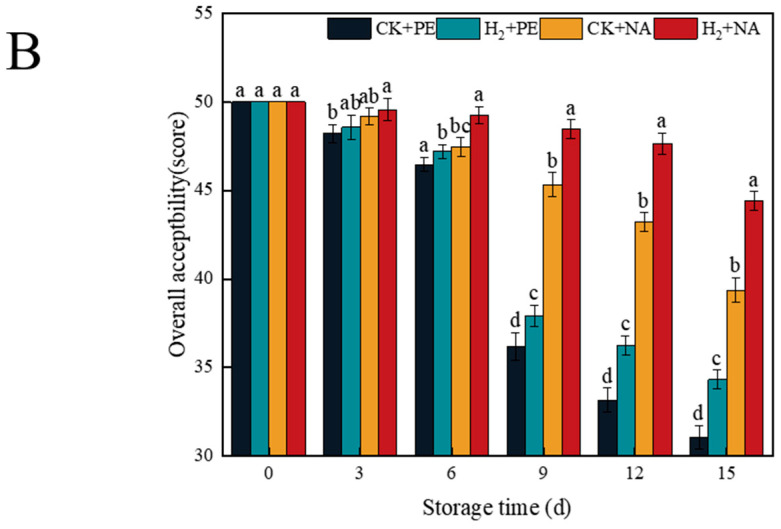
Effect of H_2_ fumigation combined with NA packaging on appearance (**A**) and overall acceptability (**B**) of *A. bisporus*. Vertical bars indicate standard error (±SE). Different letters indicate significant differences according to Duncan’s test (*p* < 0.05). All samples were kept in cold storage (4 ± 1 °C) and relative humidity of 90% for 15 d. CK + PE represents mushrooms subjected only to polyethylene film packaging treatment, H_2_ + PE represents fumigation with H_2_ and packaging with polyethylene film, CK + NA represents mushrooms subjected only to nano-film packaging treatment, and H_2_ + NA represents fumigation with H_2_ and packaged with nano-film. The same below.

The appearance of mushrooms is an important factor influencing consumers’ purchasing decision. Changes in sensory quality and overall sensory scores of samples during storage are presented in Figure 3. A decreasing trend was observed in the control and treatment groups, and obvious browning and gradual wilting began at 9 d in CK + PE and H_2_ + PE groups, with more obvious softening of the mushrooms upon completion of storage; after 15 d, the sensory score was 31.03 and 34.31, respectively, which represented a loss of commercial value. In contrast to the other three groups, the H_2_ + NA group effectively delayed browning, wilting, and softening of the fruiting bodies; maintained better sensory quality; and the difference was significant during the 9–15 d period. After 15 d of storage, the sensory score of H_2_ + NA group was 44.41, and the overall acceptability was good. The results showed that H_2_ fumigation productively delayed the deterioration in mushroom quality. In contrast to the traditional commercial PE film, H_2_ fumigation combined with NA packaging could prolong the shelf life by 4–5 d. The results indicate that H_2_ fumigation had an effect on delaying the deterioration in mushroom quality.

### 3.3. Effects of H_2_ + NA on Weight Loss and Firmness of A. bisporus

**Figure 4:**
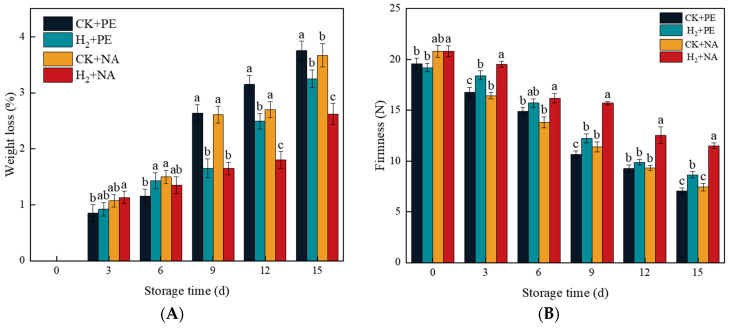
Effect of H_2_ fumigation combined with NA packaging on weight loss (**A**) and firmness (**B**) in *A. bisporus*. Different letters indicate significant differences according to Duncan’s test (*p* < 0.05).

As depicted in Figure 4A, the weight loss rate of mushrooms continue to rise during the entire storage period. When it came to 15 d of storage, the weight loss rate in the H_2_ + NA group was lower compared to that in the other three groups (*p* < 0.05), which were 68.87%, 80.62%, and 71.39% in CK + PE, H_2_ + PE, and CK + NA groups, respectively, and showed that the combination of H_2_ fumigation and NA packaging could effectively decrease the loss of mushroom quality. The firmness of the cap is among the principal factors that have an impact on the shelf life of mushrooms, reflecting the degree of softening of mushrooms during storage. As depicted in Figure 4B, the firmness of mushrooms within all treatment groups showed a downward tendency during storage, but the firmness of mushroom caps in the H_2_ + NA group was greater than that in the other three groups.

### 3.4. Effects of H_2_ + NA on L* and Browning Index of A. bisporus

**Figure 5:**
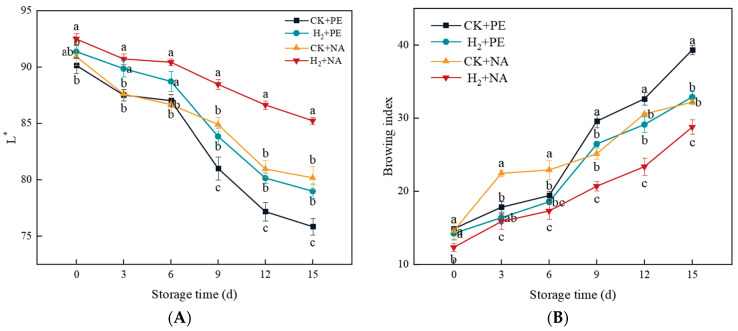
Effect of H_2_ fumigation combined with NA packaging on L* (**A**) and browning index (**B**) of *A. bisporus*. Different letters indicate significant differences according to Duncan’s test (*p* < 0.05).

The surface color of mushrooms is the most crucial factor determining consumer purchase. According to Figure 5, when the storage time was lengthened, the surface L* of mushrooms decreased and BI increased. After 9 d of storage, whiteness in the CK + PE and H_2_ + PE groups decreased compared to the H_2_ + NA group, and BI increased. On the 15th day, whiteness in the H_2_ + NA group was 1.12, 1.08, and 1.06 times that in the CK + PE, H_2_ + PE, and CK + NA groups, separately, indicating that compared with commercial PE film, H_2_ fumigation combined with NA packaging can prolong the storage time of mushrooms by 4–5 d. The BI of *A. bisporus* in the group treated with H_2_ fumigation was lower compared with the control group, and the best browning inhibition effect was achieved by H_2_ + NA packaging, which demonstrated that H_2_ fumigation combined with NA packaging could obviously retard browning of *A. bisporus* and maintain color quality.

### 3.5. Effects of H_2_ + NA on MDA in A. bisporus

**Figure 6:**
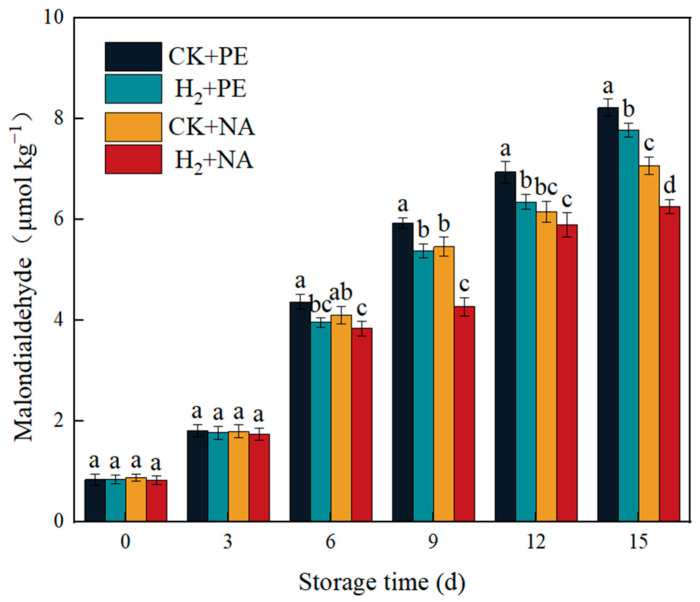
Effect of H_2_ fumigation combined with NA packaging on MDA content of *A. bisporus*. Different letters indicate significant differences according to Duncan’s test (*p* < 0.05).

The content of MDA is an important indicator used to characterize lipid peroxidation damage in edible mushroom cell membranes [26]. Therefore, the extent of membrane lipid damage can be detected through measuring the content of MDA. If the MDA content in the sample increases, this indicates the occurrence of lipid peroxidation. As depicted in Figure 6, duration storage, the content of MDA in all tested groups exhibited an upward trend, but there was a difference in the accumulation rate. The PE group had a higher increase in MDA content than the NA group, with the H_2_ + NA group having the lowest increase. While stored for 15 d, the content of MDA in H_2_ + NA group was 6.26 μmol kg^−1^, which was 31.31%, 24.12%, and 12.94% lower than that in CK + PE, H_2_ + PE, and CK + NA groups, separately. It can be seen that H_2_ fumigation combined with nano-film packaging can effectively decrease the accumulation of MDA in mushrooms and delay the aging of *A. bisporus*.

### 3.6. Effects of H_2_ + NA on O^2•−^ Generation Rate and H_2_O_2_ Content of A. bisporus

**Figure 7:**
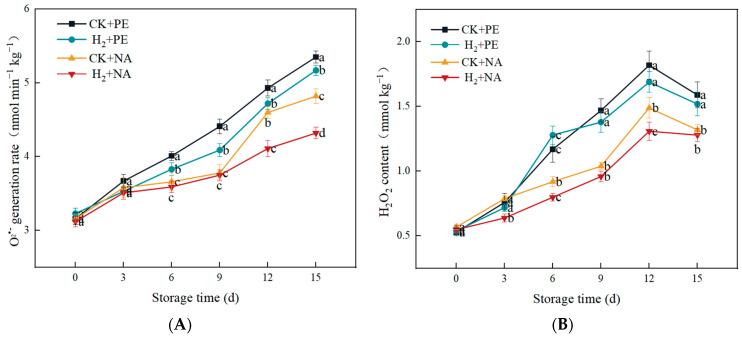
Effect of H_2_ fumigation combined with NA packaging on O^2•−^ generation rate (**A**) and H_2_O_2_ content (**B**) of *A. bisporus*. Different letters indicate significant differences according to Duncan’s test (*p* < 0.05).

The accumulation of ROS is a typical oxidative stress response. Postharvest senescence of *A. bisporus* is tightly associated with the generation and accumulation of ROS during storage, and the H_2_O_2_ content and O^2•−^ generation rate are important indicators that characterize ROS. As shown in Figure 7, during storage, the O^2•−^ generation rate in every group exhibited a rising trend, with the CK + PE group having the highest O^2•−^ generation rate, while the H_2_ + NA group had a relatively slow O^2•−^ generation rate. The H_2_O_2_ content showed an increasing trend and then decreased, showing a decrease after 12 d of storage, and the H_2_O_2_ content in H_2_ + NA was continuously at a low level. In conclusion, H_2_ + NA could effectively decrease the rate of O^2•−^ generation and the content of H_2_O_2_, which indicated that H_2_ fumigation + NA packaging could significantly inhibit the generation of ROS, which had a positive effect on the removal of ROS, maintaining them at a lower level and reducing damage in the cellular tissues of mushrooms caused by the accumulation of ROS.

### 3.7. Effects of H_2_ + NA on SOD and CAT Activities of A. bisporus

**Figure 8:**
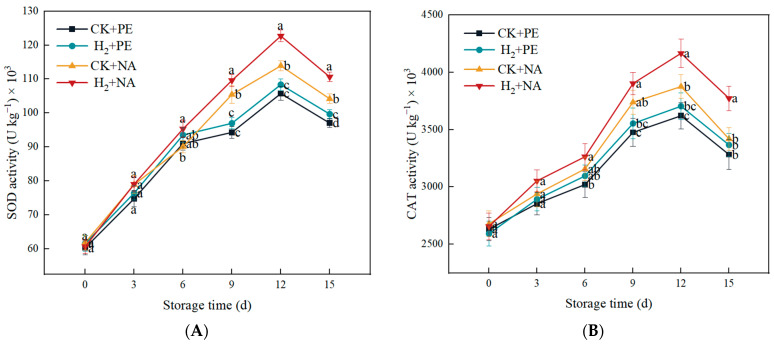
Effect of H_2_ fumigation combined with NA packaging on SOD activity (**A**) and CAT activity (**B**) of *A. bisporus*. Different letters indicate significant differences according to Duncan’s test (*p* < 0.05).

SOD is a key enzyme that protects plant cells from oxidative damage, catalyzing the decomposition of H_2_O_2_ and resisting traumatic stress caused by ROS [27]. As shown in Figure 8A, the SOD activity showed an increasing tendency then reduced at the period of storage. Compared with the CK + PE and H_2_ + PE groups, the CK + NA and H_2_ + NA groups effectively slowed the decline in mushroom SOD activity. On the 15th day, the SOD activity in H_2_ + NA was 110.69 U kg^−1^ × 10^3^, which was 1.14, 1.11, and 1.06 times that in the CK + PE, H_2_ + PE, and CK + NA groups, separately; meanwhile, SOD in the H_2_ + NA group was kept at a higher level throughout the storage process.

CAT is a key antioxidant enzyme that clears ROS and protects plants from oxidative damage. By catalyzing the decomposition of H_2_O_2_ into O_2_ and H_2_O, CAT reduces ROS accumulation, thereby reducing cell membrane damage and maintaining normal cell function, thus extending the shelf life of fruit and vegetables. As depicted in Figure 8B, as storage time extended, mushroom CAT activity in all groups showed an increasing trend and then decreased, which was basically the same as the trend in H_2_O_2_ content; CAT activity in each group arrived a peak on the 12th day, and at end storage, the activity of CAT in H_2_ + NA was 1.15, 1.12, and 1.10 times that in CK + PE, H_2_ + PE, and CK + NA groups, separately. In conclusion, H_2_ fumigation combined with NA packaging effectively delayed the decline in CAT activity in mushrooms.

### 3.8. Effects of H_2_ + NA on Flavonoid Content and Total Phenolic Content in A. bisporus

**Figure 9:**
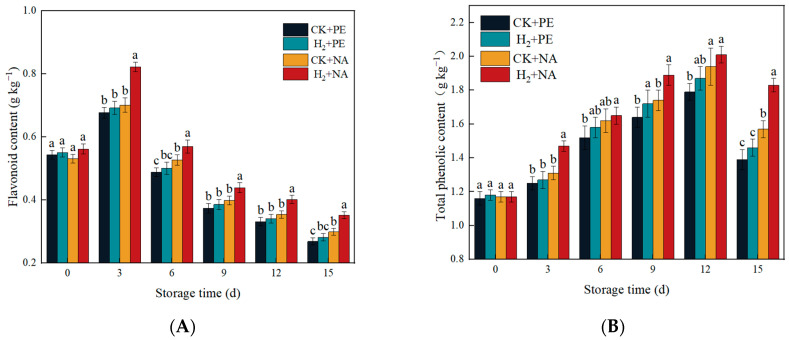
Effect of H_2_ fumigation combined with NA packaging on flavonoid content (**A**) and total phenolic content (**B**) in *A. bisporus*. Different letters indicate significant differences according to Duncan’s test (*p* < 0.05).

Flavonoids are important bioactive substances in *A. bisporus*, and are secondary metabolites, with antioxidant activity [28], microbial growth inhibition, anti-tumor effects, and other properties. According to Figure 9A, during storage, the flavonoid content in mushrooms showed an initial increasing trend and then decreased. The flavonoid content in the H_2_ + NA group remained at a relatively high level, indicating that H_2_ + NA treatment can reduce the consumption of flavonoids during postharvest storage and has a certain effect on maintaining the active substances in mushrooms. This may be due to the fact that nano-film packaging can reduce oxygen permeation, decrease the oxidation reaction of mushrooms, and improve the retention rate of flavonoids.

Phenolic compounds are secondary metabolites with antioxidant capacity produced during the phenylpropane metabolism of plants, which have an important impact in the postharvest stress resistance of fruit and vegetables [29]. As can be observed from Figure 9B, the total phenolic content in H_2_ + NA was always higher than that in the other three groups during the storage of mushrooms, and the total phenolic content in each group of mushrooms exhibited an overall upward trend at first and then declined during the storage process. This may be due to oxidative stress during the initial period of storage, which accelerates the synthesis of phenolic substances in the mushrooms during storage to resist environmental stress. On the 15th day, the total phenolic content in H_2_ + NA was 1.83 g kg^−1^, which was 1.32, 1.25, and 1.17 times that in CK + PE, H_2_ + PE, and CK + NA, respectively. It concluded that H_2_ fumigation + NA packaging played a positive role in the accumulation of total phenols in mushrooms.

### 3.9. Effects of H_2_ + NA on Total Aerobic Plate Count in A. bisporus

**Figure 10:**
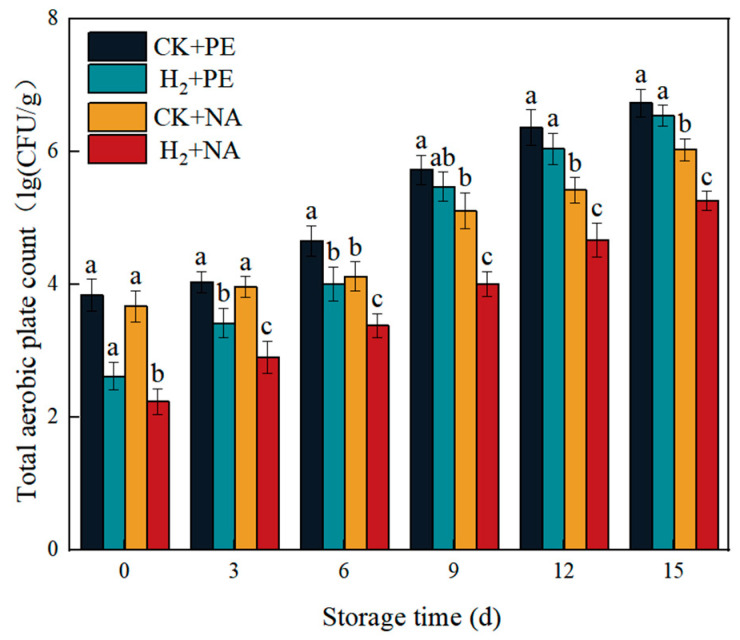
Effect of H_2_ fumigation combined with NA packaging on total aerobic plate count of *A. bisporus*. Different letters indicate significant differences according to Duncan’s test (*p* < 0.05).

The invasion of bacteria can cause the decay of the fruiting bodies of edible fungi. As shown in Figure 10, during the entire storage process, the total number of colonies in each group showed an upward trend. The total number of colonies in the CK + PE group and the H_2_ + PE group were higher than those in the CK + NA group and the H_2_ + NA group, respectively. Among them, the total number of colonies in the H_2_ + NA group was always lower than that in the other three groups, indicating that H_2_ fumigation combined with NA packaging has a good antibacterial effect.

### 3.10. Effects of H_2_ + NA on Protein Content in A. bisporus

**Figure 11:**
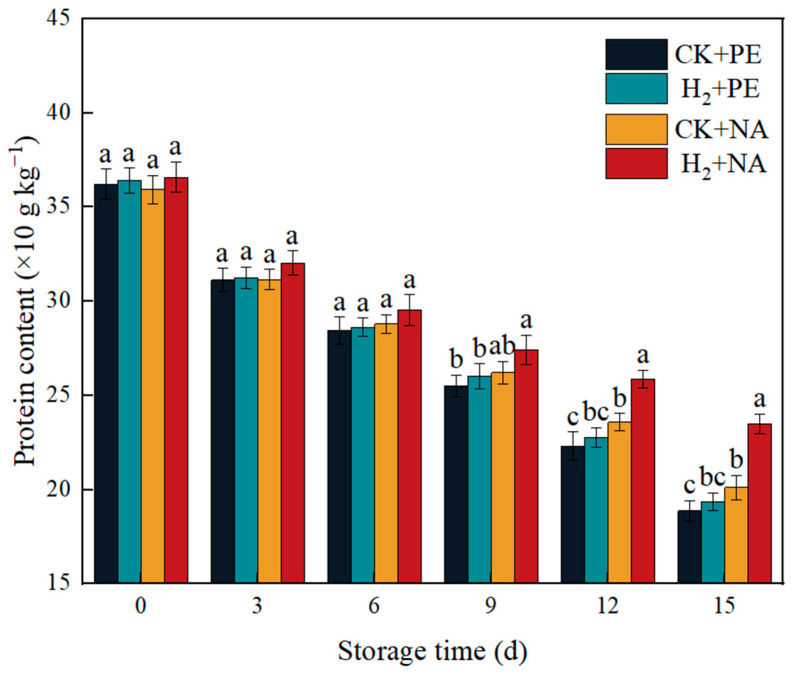
Effect of H_2_ fumigation combined with NA packaging on protein content in *A. bisporus*. Different letters indicate significant differences according to Duncan’s test (*p* < 0.05).

Protein is one of the important nutritional indicators of edible mushrooms. Figure 11 reflects the changes in protein content of mushrooms during storage. The protein content of each group showed a decreasing tendency throughout the storage; this was due to the lack of a nitrogen source after harvesting of edible mushrooms and the presence of proteases in the substrate, which led to the decomposition of protein into peptides, peptones, and amino acids. The difference in protein content in the mushrooms in each group was not significant during 0–6 d in the pre-storage period; browning and autolysis began to appear in the CK + PE and H_2_ + PE groups at 9 d, the rate of protein degradation was accelerated, and protein retention was higher in the nano-packaging groups CK + NA and H_2_ + NA, of which the protein content in H_2_ + NA group was constantly at a higher level. It can be seen that H_2_ fumigation + NA packaging effectively slowed the degradation of proteins and maintained the nutritional quality of the mushrooms well.

## 4. Discussion

Due to the unique antioxidant properties and reducing ability of H_2_, it has a significant impact on plant seed germination [30], growth and development [31], yield increase and quality improvement [32], stress tolerance [33], and agricultural product preservation [34]. At present, research on the application of H_2_ treatment in the field of agriculture has received widespread attention [35], and the use of H_2_-rich water immersion treatment has increased, but the application of H_2_ fumigation treatment in the preservation of agricultural products is still unreported, and the mechanism of its action is still unclear.

In this research, we delved the impact of composite preservation technology with H_2_ fumigation combined with nano-film packaging on the postharvest deterioration and antioxidant capacity of mushrooms. The hardness, whiteness, browning, weight loss, nutritional quality, ROS content, and antioxidant enzyme activity of *A. bisporus* were dynamically measured as an important basis for evaluating the postharvest deterioration in mushroom quality. The findings revealed that disparities were present in the preservation effect of mushrooms under the four packaging methods used. In terms of the overall quality change and antioxidant capacity of mushrooms during the storage, the overall quality of *A. bisporus* packaged in NA film was better than that packaged in ordinary PE cling film, and the quality of H_2_ fumigated mushrooms was better than that of untreated mushrooms. The storage test confirmed that H_2_ fumigation + NA packaging effectively postponed the occurrence of softening and browning of *A. bisporus*, reduced the rate of quality loss, and inhibited mushroom opening.

Previous studies have shown that oxidative damage in fruit and vegetable tissues is intensified during ripening and aging owing to ROS accumulation [36], which in turn destroys the cellular structure and further exacerbates oxidative damage [37], thus accelerating the aging process. An imbalance between ROS production and scavenging leads to the accumulation of ROS, which can decrease the storage quality and marketability of fruit and vegetables [38]. Research has shown that after treatment with hydrogen rich water, the expression of antioxidant enzyme related genes such as SOD and CAT in *Hypsizygus marmoreus* is upregulated during storage, consistent with changes in antioxidant enzyme activity, thereby reducing ROS accumulation [39]. The outcomes of this study demonstrated that H_2_ fumigation for 2 h combined with NA packaging treatment effectively inhibited the production of ROS in *A. bisporus*, and at the same time increased the activities of SOD and CAT in mushrooms. This further verified that H_2_ fumigation combined with NA packaging could activate the antioxidant defense system of mushrooms to control the ROS content, and thus reduce oxidative damage and slow down aging.

The H_2_ fumigation combined with NA composite preservation technology developed in this study has comparable preservation effect, operability, and cost compared to other commercial MAP preservation technologies such as ozone fumigation [2,19], and does not produce ozone odor residue, making it more suitable for implementing this method on an industrial scale.

## 5. Conclusions

In this presenting study, the composite preservation technology combining H_2_ fumigation treatment and NA packaging was applied in the postharvest preservation of *A. bisporus*. It showed that H_2_ fumigation for 2 h combined with NA packaging effectively delayed the quality deterioration and activated the antioxidant defense system in the mushrooms, which reduced oxidative damage by inhibiting the accumulation of ROS and delayed aging of the mushroom body with a good preservation effect. Compared with the control group packed with PE film, H_2_ fumigation combined with NA packaging prolonged the shelf life of mushrooms by 4–5 d. These findings suggest that this treatment may be a simple, convenient, safe, cheaper, and practical solution for prolonging the shelf life of button mushrooms and may also be beneficial for enhancing the postharvest quality of other edible fungi, with high commercial potential and broad industrialization prospects.

## Figures and Tables

**Table 1:** Sensory quality evaluation standard of *A. bisporus*.

Score	Color (S1)	Cap Shape (S2)	Off-Odor (S3)	Texture Stretchy (S4)	Consumer Acceptance (S5)	Sensory Score
10–8	White	Closed	No	Stretchy	Intense	—
8–6	Slight browning	Slightly open	Slight	Slight soft	Acceptable	—
6–4	Mild browning	Half open	Obvious	Mild soft	Discount	—
<4	Heavy browning	Totally open	Severe	Severe soft	Unacceptable	—

Note: Sensory score = S1 + S2 + S3 + S4 + S5. (Evaluators blinded to the treatments).

**Table 2:** Effects of H_2_ fumigation at different times combined with NA packaging on sensory scores, browning index, and weight loss of *A. bisporus.* Different letters indicate significant differences according to Duncan’s test (*p* < 0.05).

		Time/d					
Index		0	3	6	9	12	15
Sensory score	CK	50.00 ± 0.00 ^a^	49.18 ± 0.47 ^a^	47.47 ± 0.52 ^b^	45.33 ± 0.68 ^c^	43.21 ± 0.54 ^d^	39.37 ± 0.71 ^d^
	1 h	50.00 ± 0.00 ^a^	49.23 ± 0.39 ^a^	47.84 ± 0.61 ^b^	46.59 ± 0.44 ^b^	44.36 ± 0.72 ^c^	41.22 ± 0.59 ^c^
	2 h	50.00 ± 0.00 ^a^	49.58 ± 0.63 ^a^	49.27 ± 0.48 ^a^	48.45 ± 0.53 ^a^	47.64 ± 0.61 ^a^	44.41 ± 0.52 ^a^
	3 h	50.00 ± 0.00 ^a^	49.36 ± 0.41 ^a^	49.01 ± 0.34 ^a^	47.97 ± 0.56 ^a^	46.51 ± 0.52 ^b^	42.72 ± 0.67 ^b^
Browning index	CK	14.58 ± 1.06 ^a^	22.46 ± 0.44 ^a^	22.91 ± 1.31 ^a^	25.13 ± 0.72 ^a^	30.57 ± 0.23 ^a^	32.18 ± 0.3 ^a^
	1 h	15.22 ± 0.54 ^a^	17.18 ± 0.18 ^b^	18.15 ± 1.25 ^b^	20.53 ± 1.31 ^b^	24.75 ± 1.74 ^b^	30.10 ± 0.46 ^bc^
	2 h	12.33 ± 0.55 ^b^	15.86 ± 1.03 ^b^	17.31 ± 1.15 ^b^	20.7 ± 0.67 ^b^	23.37 ± 1.20 ^b^	28.78 ± 1.01 ^c^
	3 h	14.21 ± 0.47 ^a^	16.38 ± 0.86 ^b^	18.30 ± 1.30 ^b^	22.04 ± 1.13 ^b^	25.74 ± 2.21 ^b^	32.12 ± 1.30 ^ab^
Weight loss rate	CK	0.00 ± 0.00 ^a^	1.07 ± 0.11 ^a^	1.50 ± 0.12 ^ab^	2.61 ± 0.15 ^a^	2.70 ± 0.14 ^a^	3.67 ± 0.21 ^a^
	1 h	0.00 ± 0.00 ^a^	1.21 ± 0.14 ^a^	1.40 ± 0.16 ^b^	1.79 ± 0.13 ^b^	2.34 ± 0.22 ^b^	3.65 ± 0.18 ^a^
	2 h	0.00 ± 0.00 ^a^	1.13 ± 0.11 ^a^	1.35 ± 0.15 ^b^	1.65 ± 0.11 ^b^	1.80 ± 0.15 ^c^	2.62 ± 0.19 ^b^
	3 h	0.00 ± 0.00 ^a^	1.17 ± 0.12 ^a^	1.73 ± 0.15 ^a^	2.73 ± 0.13 ^a^	2.96 ± 0.15 ^a^	3.43 ± 0.18 ^a^

## Data Availability

The original contributions presented in the study are included in the article, further inquiries can be directed to the corresponding author.

## References

[1] Siwulski M., Budka A., Rzymski P., Gąsecka M., Kalač P., Budzyńska S., Magdziak Z., Niedzielski P., Mleczek P., Mleczek M. (2019). Worldwide basket survey of multielemental composition of white button mushroom *Agaricus bisporus*. Chemosphere.

[2] Wang T., Yun J., Zhang Y., Bi Y., Zhao F., Niu Y. (2021). Effects of ozone fumigation combined with nano-film packaging on the postharvest storage quality and antioxidant capacity of button mushrooms (*Agaricus bisporus*). Postharvest Biol. Technol..

[3] Qu T., Li B., Huang X., Li X., Ding Y., Chen J., Tang X. (2020). Effect of peppermint oil on the storage quality of white button mushrooms (*Agaricus bisporus*). Food Bioprocess Technol..

[4] Esmaeili Y., Zamindar N., Mohammadi R. (2023). The effect of polypropylene film containing nano-hydroxyapatite on physicochemical and microbiological properties of button mushrooms (*Agaricus bisporus*) under modified atmosphere packaging. J. Food Meas. Charact..

[5] Vunduk J., Kozarski M., Djekic I., Tomaevi I., Klaus A. (2021). Effect of modified atmosphere packaging on selected functional characteristics of *Agaricus bisporus*. Eur. Food Res. Technol..

[6] Jaworska G., Sidor A., Pycia K., Jaworska-Tomczyk K., Surówka K. (2020). Packaging method and storage temperature affects microbiological quality and content of biogenic amines in *Agaricus bisporus* fruiting bodies. Food Biosci..

[7] Yu Y., Zhang H., Xing H., Cui N., Liu X., Meng X., Wang X., Fan L., Fan H. (2023). Regulation of growth and salt resistance in cucumber seedlings by hydrogen-rich water. J. Plant Growth Regul..

[8] Qiu P., Liu Y., Zhang J. (2019). Recent advances in studies of molecular hydrogen against sepsis. Int. J. Biol. Sci..

[9] Hancock J.T., Russell G., Stratakos A.C. (2022). Molecular hydrogen: The postharvest use in fruits, vegetables and the floriculture industry. Appl. Sci..

[10] Liu S., Zha Z., Chen S., Tang R., Zhao Y., Lin Q., Duan Y., Wang K. (2023). Hydrogen-rich water alleviates chilling injury-induced lignification of kiwifruit by inhibiting peroxidase activity and improving antioxidant system. J. Sci. Food Agric..

[11] Liu F., Li J., Liu Y. (2016). Molecular hydrogen can take part in phytohormone signal pathways in wild rice. Biol. Plant..

[12] Zhang H., Wu X., Liu X., Yao Y., Liu Z., Wei L., Hou X., Gao R., Li Y., Wang C. (2023). Hydrogen gas improves the postharvest quality of Lanzhou lily (*Lilium davidii* var. *unicolor*) bulbs. Plants.

[13] Jiang K., Kuang Y., Feng L., Liu Y., Wang S., Du H., Shen W. (2021). Molecular hydrogen maintains the storage quality of Chinese chive through improving antioxidant capacity. Plants.

[14] Wang Y., Wang J., Kuang Y., Shen W. (2021). Packaging with hydrogen gas modified atmosphere can extend chicken egg storage. J. Sci. Food Agric..

[15] Jiang K., Zhang Y., Cai C., Lin W., Li L., Shen W. (2023). Hydrogen-based modified atmosphere packaging delays the deterioration of dried shrimp (*Fenneropenaeus chinensis*) during accelerated storage. Food Control.

[16] Xing Y., Li X., Zhang L., Xu Q., Che Z., Li W., Bai Y., Li K. (2012). Effect of TiO_2_ nanoparticles on the antibacterial and physical properties of polyethylene-based film. Prog. Org. Coat..

[17] Brito S.D.C., Bresolin J.D., Sivieri K., Ferreira M.D. (2019). Low-density polyethylene films incorporated with silver nanoparticles to promote antimicrobial efficiency in food packaging. Food Sci. Technol. Int..

[18] Perez-Esteve E., Bernardos A., Martínez-Máñez R., M Barat J. (2013). Nanotechnology in the development of novel functional foods or their package. An overview based in patent analysis. Recent Pat. Food Nutr. Agric..

[19] Wang B., Yun J., Ye C., Xu S., Guo W., Zhao F., Qu Y., Bi Y. (2024). A novel polyethylene nanopackaging combined with ozone fumigation delayed the browning and softening of *Agaricus bisporus* during postharvest storage. Postharvest Biol. Technol..

[20] Lin Q., Lu Y., Zhang J., Liu W., Guan W., Wang Z. (2017). Effects of high CO_2_ in-package treatment on flavor, quality and antioxidant activity of button mushroom (*Agaricus bisporus*) during postharvest storage. Postharvest Biol. Technol..

[21] Li X., Zhang Y., Zhang Y., Liu Y., Gao Z., Zhu G., Xie Y., Mowafy S.G. (2022). Relative humidity control during shiitake mushroom (*Lentinus edodes*) hot air drying based on appearance quality. J. Food Eng..

[22] Kotwaliwale N., Bakane P., Verma A. (2007). Changes in textural and optical properties of oyster mushroom during hot air drying. J. Food Eng..

[23] Zheng H., Liu W., Liu S., Liu C., Zheng L. (2019). Effects of melatonin treatment on the enzymatic browning and nutritional quality of fresh-cut pear fruit. Food Chem..

[24] Gao M., Feng L., Jiang T. (2014). Browning inhibition and quality preservation of button mushroom (*Agaricus bisporus*) by essential oils fumigation treatment. Food Chem..

[25] Tan X., Fan Z., Zeng Z., Shan W., Kuang J., Lu W., Su X., Tao N., Lakshmanan P., Chen J. (2021). Exogenous melatonin maintains leaf quality of postharvest Chinese flowering cabbage by modulating respiratory metabolism and energy status. Postharvest Biol. Technol..

[26] Zhou R., Li Y., Yan L., Xie J. (2011). Effect of edible coatings on enzymes, cell-membrane integrity, and cell-wall constituents in relation to brittleness and firmness of Huanghua pears (*Pyrus pyrifolia* Nakai, cv. Huanghua) during storage. Food Chem..

[27] Guo Y., Chen X., Gong P., Guo J., Deng D., He G., Ji C., Wang R., Long H., Wang J. (2022). Effect of shiitake mushrooms polysaccharide and chitosan coating on softening and browning of shiitake mushrooms (*Lentinus edodes*) during postharvest storage. Int. J. Biol. Macromol..

[28] Li F., Hu Y., Shan Y., Liu J., Ding X., Duan X., Zeng J., Jiang Y. (2022). Hydrogen-rich water maintains the color quality of fresh-cut Chinese water chestnut. Postharvest Biol. Technol..

[29] Wang C., Meng X. (2016). Effect of 60Co γ-irradiation on storage quality and cell wall ultra-structure of blueberry fruit during cold storage. Innov. Food Sci. Emerg. Technol..

[30] Huang P., Li C., Liu H., Zhao Z., Liao W. (2021). Hydrogen gas improves seed germination in cucumber by regulating sugar and starch metabolisms. Horticulturae.

[31] Zeng J., Yu H. (2022). Integrated metabolomic and transcriptomic analyses to understand the effects of hydrogen water on the roots of *Ficus hirta Vahl*. Plants.

[32] Cheng P., Wang J., Zhao Z., Kong L., Lou W., Zhang T., Jing D., Yu J., Shu Z., Huang L. (2021). Molecular hydrogen increases quantitative and qualitative traits of rice grain in field trials. Plants.

[33] Xie Y., Mao Y., Lai D., Zhang W., Shen W. (2012). H_2_ enhances *Arabidopsis* salt tolerance by manipulating ZAT10/12-Mediated antioxidant defence and controlling sodium exclusion. PLoS ONE.

[34] Dong W., Shi L., Li S., Xu F., Yang Z., Cao S. (2023). Hydrogen-rich water delays fruit softening and prolongs shelf life of postharvest okras. Food Chem..

[35] Hou X., Qi N., Wang C., Li C., Huang D., Li Y., Wang N., Liao W. (2021). Hydrogen-rich water promotes the formation of bulblets in *Lilium davidii* var. unicolor through regulating sucrose and starch metabolism. Planta.

[36] Marangoni A.G., Palma T., Stanley D.W. (1996). Membrane effects in postharvest physiology. Postharvest Biol. Technol..

[37] Jiang T., Zheng X., Li J., Jing G., Cai L., Ying T. (2011). Integrated application of nitric oxide and modified atmosphere packaging to improve quality retention of button mushroom (*Agaricus bisporus*). Food Chem..

[38] Sharma P., Jha A.B., Dubey R.S., Pessarakli M. (2012). Reactive oxygen species, oxidative damage, and antioxidative defense mechanism in plants under stressful conditions. J. Bot..

[39] Chen H., Zhang J., Hao H., Feng Z., Chen M., Wang H., Ye M. (2017). Hydrogen-rich water increases postharvest quality by enhancing antioxidant capacity in *Hypsizygus marmoreus*. AMB Express.

